# Electrophoretic deposition and characterization of self-doped SrTiO_3_ thin films

**DOI:** 10.3906/kim-2007-13

**Published:** 2021-04-28

**Authors:** Naeimeh Sadat PEIGHAMBARDOUST, Umut AYDEMİR

**Affiliations:** 1 Koç University Boron and Advanced Materials Application and Research Center, İstanbul Turkey; 2 Department of Chemistry, Koç University, İstanbul Turkey

**Keywords:** Blue SrTiO_3_, electrophoretic deposition, electrochemical reactions, oxygen vacancy, photocatalysis

## Abstract

Herein, titanium (Ti^3+^) self-doped strontium titanate (SrTiO_3_), so-called blue SrTiO_3_, with a bandgap of 2.6 eV and favorable photocatalytic characteristics was fabricated through a facile and effective method. For electrochemical investigations, the electrophoretic deposition was applied to produce SrTiO_3_ thin films on (fluorine-doped tin oxide) FTO conductive substrates. The electrophoretic voltage of 20 V and a process duration of 10 min were optimized to reach transparent and uniform coatings on FTO. The blue SrTiO_3_ reveals lower resistance (charge transfer resistance of 6.38 Ω cm^-2^) and higher electron mobility (current density value of 0.25 mA cm^-2^) compared to a pure SrTiO_3_ electrode. These findings may provide new insights for developing high-performance visible light photocatalysts.

## 1. Introduction

Hydrogen energy has recently attracted a great deal of attention due to environmental issues of using fossil fuels. Hydrogen can be produced from various sources, such as methanol, natural gas, coal, and solar cells [1−3]. It comes as no surprise that a large amount of hydrogen is supplied by fossil fuels [1]. Pure hydrogen generation is one of the critical challenges that push researchers towards hydrogen production from water by using solar irradiation. One of the most important schemes to tackle energy problems is by a water splitting reaction using an efficient photocatalyst [4]. Water splitting can directly produce clean hydrogen from water couples with renewable solar energy [5−7]. Solar irradiation, along with an efficient photocatalyst can decompose water into H_2_ and O_2_ [8]. Different forms of solar energy such as heat, light, and electricity can be used to produce hydrogen from water. Among them, light is the encouraging solar path due to suppressed thermal transportation [9].

Regarding the mentioned merits, water electrolysis by photocatalysis plays a pivotal role in clean energy production. During photocatalytic water splitting, a semiconductor adsorbs light with an energy that is larger than its bandgap, leading to the generation of electron and hole pairs through the conduction and valence bands, respectively. The photoinduced electrons reduce water to hydrogen at an interface between water and the photocatalyst. On the other side, holes oxidize water to oxygen, resulting in overall water splitting [10,11].

Photocatalytic semiconductors have been potentially used for water splitting. The photocatalytic properties are strongly affected by the potential of the band structure, charge separation, lifetime, and mobility of photogenerated electron and holes. These properties are provoked by bulk and surface properties of materials such as crystallinity and surface-active sites [12]. To harvest visible light irradiation, new photocatalysts are designed for the splitting of water based on their band structure positions. According to the previous studies, photocatalytic reactions can occur in three processes: 1) photo absorption and formation of electron-hole pair, 2) charge separation, and photogenerated carrier migrations, and 3) the surface chemical reactions for hydrogen evolution [13].

The TiO_2_ photocatalyst, as the most well-studied photocatalyst, contains a wide bandgap of 3.0 eV and an insufficient conduction band level for hydrogen generation. It needs UV light and an external bias to be induced to decompose water. The bandgap of photocatalysts should be narrower than 3.0 eV to harvest visible light [14−16]. Moreover, an efficient photocatalyst for water splitting should have proper band positions. For more efficient water decomposition, the conduction band must be located above the potential of water reduction (H^+^/H_2_). On the other hand, the valence band must be located below the water oxidation potential. To date, a wide variety of semiconductors have been employed as photocatalysts for water splitting such as TiO_2_ [16], SrTiO_3_ [17], Ta_2_O_5_ [18], K_2_La_2_Ti_3_O_10_ [19], Nb_2_O_5_ [20], Fe_2_O_3_ [21], and BiVO_4_ [22].

Among the variety of semiconductors as photocatalysts, perovskite-type oxides are favorable thanks to their peculiar properties. Lately, ABO3 perovskites have been introduced as conventional photocatalytic materials. SrTiO_3_ is one of the most promising photocatalytic perovskites because of its high resistance against photocorrosion, high thermal stability, low cast, and exceptional optical and electrical properties. The conduction and valence band potentials of SrTiO_3_ are positioned in larger negative domains than TiO_2_, making SrTiO_3_ a more efficient photocatalyst in water splitting without applying any bias [12,23,24].

SrTiO_3_ is synthesized through different methods such as a solid-state reaction [12] and solvothermal method [25]. In this sense, the solvothermal method is more advantageous owing to proper control of stoichiometry, homogeneity, as well as shape and morphology of powders. On the contrary, the solid-state method fails to fulfill the desired properties. SrTiO_3_ suffers from its wide bandgap. Various strategies have been allotted to reduce the bandgap and consequently enhance visible light harvesting. Doped SrTiO_3_, such as Cr, Ta-doped SrTiO_3_ [26], and Rh-doped SrTiO_3_ [27] have been introduced to reduce the SrTiO_3_ bandgap. Moreover, the coupling of doped SrTiO_3_ with a second photocatalyst such as Pt has been applied to generate more H_2_ and O_2_ in the presence of visible light irradiation [28]. 

Although many attempts have been made on expanding SrTiO_3_ response to the visible region, including metal and nonmetal doping, combining with semiconductors and so on, harvesting visible irradiation remains a formidable challenge. It should be mentioned that dopants act as trap sites for recombination of electron-hole. For more efficient photocatalysis, the recombination rate should be diminished. Recently, reduced SrTiO_3_ has been reported for the decomposition of water into H_2_ and O_2_ under visible light illumination. Efforts have been made on the decomposition rate under visible illumination [29,30]. Therefore, the performance of SrTiO_3_ is affected by lattice structure and surface electronic as a consequence of the presence of the different Ti coordination configuration and oxidation states on the surface [30,31]. Reduced SrTiO_3_ plays an important role in enhancing photocatalytic activity. Surface oxygen vacancies formed during the SrTiO_3_ reduction process act as adsorption sites for the photo-induced electron-hole [30,32]. Through these sites, the charge can move toward surface compounds, diminish the recombination rate of photoinduced charges, and thereby improve the photocatalytic activity. On the other hand, bulk oxygen vacancies act as trap sites for photoinduced charges, leading to a decrease in the activity. However, controlling the surface oxygen vacancies is vital for enhancing photocatalytic performance [30,31].

It is worth noting that the powder form of photocatalysts has low crystallinity and it is often difficult to recover from the solution. Besides, in the bulk form, it is nearly impossible to distinguish the photocarrier dynamic effect. In contrast, thin-film photocatalytic materials have good crystallinity and numerous catalytic active surface [16].

All in all, modification of a reduced SrTiO_3_ bandgap under visible light is necessary to accomplish an active photocatalyst. In this study, as an initial step, the bandgap modification of reduced blue SrTiO_3_ (STO) was carried out. Acquiring a uniform blue SrTiO_3_ thin film on a conductive glass is the primary motivation of this research. In doing so, the electrophoretic deposition was conducted on as-synthesized blue STO powders to obtain a thin film of perovskite photoelectrodes to be used in photocatalytic water splitting applications. 

## 2. Material and methods

### 2.1. SrTiO_3_ powders

The STO powders were prepared by hydrothermal synthesis. Tetrabutyltitanate (Ti(OCH_2_CH_2_CH_2_CH3)4), strontium nitrate (Sr(NO_3_)_2_), sodium hydroxide (NaOH), ethanol, and deionized water were used as precursors. For this, 3.7 mL of tetrabutyltitanate was added to 50 mL ethanol and 2.304 g Sr(NO_3_)_2_ was dissolved in 20 mL deionized water, respectively at room temperature. A stirring magnet was used during the processes for 30 min. After that, the Sr(NO_3_)_2_ solution was quickly added to tetrabutyltitanate solution, followed by 10 mL of 5 M NaOH addition. After vigorous stirring for 30 min, the resulting mixture was directly transferred to a 100 mL Teflon lined stainless-steel autoclave and kept at 180 °C for 24 h. White solid particles were collected by filtration and then washed with ethanol and deionized water; thus, STO powders were gained after drying at 70 °C for 2 h. To remove SrCO_3_, the as-prepared white powders were leached by 0.5 M HNO3, washed with ethanol and deionized water, and dried at 70 °C for 2 h.

### 2.2. Blue SrTiO_3_ powders

As-prepared STO powder (25 mmol) of NaBH_4_ (25 mmol) were mixed in an agate mortar and ground for 30 min. Then, the mixture was put in a porcelain boat, placed in a tubular furnace, heated from room temperature to 350, 400, and 450 °C under the Ar+/H_2_ atmosphere at a heating rate of 10 °C min^-1^, and then held at the designed temperature for 60 min. After naturally cooling, the colored STO sample was obtained by simply washing with deionized water and ethanol several times to remove the unreacted NaBH_4_ and drying at 80 °C.

### 2.3. Electrodeposition of blue SrTiO_3_ powder on FTO glass

The electrophoretic deposition was used to deposit the blue STO nanoparticles on a fluorine-doped tin oxide (FTO) transparent conductive glass substrate, described as follows: 50 mg of STO nanoparticles were dispersed in a 40 mL of 0.2 mg/mL I_2_/acetone solution under ultrasonic treatment. A two-electrode process was used to deposit the samples at the applied potentials of 20, 30, 40, and 50 V for 10, 20, 30, and 40 min. The electrolyte was agitated by magnet stirring at 600 rpm. The FTO glass substrates with the coated area about 1 × 2 cm^2^ were used for both electrodes. Then, the deposited electrode was dried at 200 °C under the Ar^+^/H_2_ atmosphere for 30 min to remove I_2_ residues.

### 2.4. Characterization

The crystal structure and the phase purity analysis of the samples were carried out by X-ray diffraction (XRD, Rigaku Mini Flex 600, Tokyo, Japan) with Cu Kα (λ = 1.5418 Å) radiation (40 kV voltage and 15 mA). High-resolution X-ray photoelectron spectroscopy (XPS) was performed with a Thermo K-Alpha (Thermo Fisher Scientific Inc., Massachusetts, USA) with an Al Kα source. The fittings were accomplished with Avantage software. All peaks were corrected with respect to the C1s peak at 284.5 eV. For the analysis of the microstructure and surface morphology coupled with chemical composition, field emission scanning electron microscopy (FESEM) was employed by the Zeiss Ultra Plus field emission scanning electron microscope (Carl Zeiss, Oberkochen, Germany ), using accelerating voltages of 5 and 12 kV, separately. 

Electrochemical measurements of the prepared electrocatalysts were conducted in a 0.1 M KH_2_PO_4_ solution using a three-electrode cell equipped with Ag/AgCl as a reference electrode and Pt wire as a counter electrode. All experiments were evaluated at room temperature using a VersaSTAT Potentiostat Galvanostat (AMETEK Scientific Instruments, Wokingham, UK). For electrochemical measurements, each sample underwent open circuit potential (OCP) measurements for 900 s in order to stabilize in the electrolyte. Then electrochemical impedance spectroscopy (EIS) was used to evaluate the charge transfer process as the critical factor determining the electrocatalytic activity on the electrodes. The frequency range was selected between 100 kHz and 0.1 Hz with a 10 mV rms sinusoidal modulation at OCP potential. 

## 3. Results and discussion

**Figure 1:**
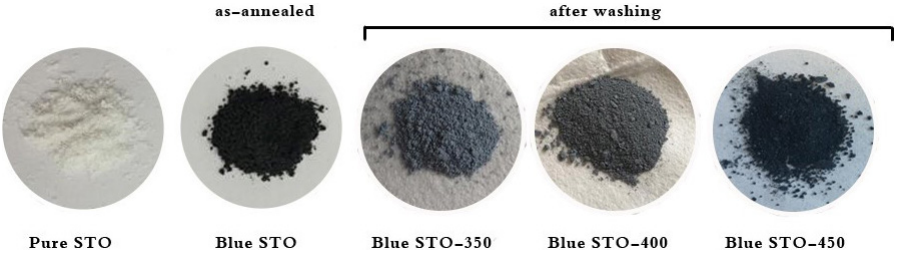
The macroimages of as-synthesized SrTiO3 (pure STO), as annealed SrTiO3 with NaBH4 (blue STO), annealed SrTiO3 with NaBH_4_ at temperatures of 350, 400, and 450 °C (blue STO-350, blue STO-400, and blue STO-450, respectively), washed with water and ethanol.

In the method described above, blue STO is produced by means of NaBH_4_ as an oxygen scavenger. In this regard, active hydrogen can be generated following NaBH_4_ decomposition. It should be highlighted that the active hydrogen acts more reactive than hydrogen. Therefore, it can remove the oxygen atoms from the surface of the STO. Thus, oxygen vacancies are produced at low temperature and short time. Figure 1 reveals the color of STO turned from white to blue. Pure STO shows as-synthesized pure SrTiO_3_. After the NaBH_4_ reaction with STO, blue STO is obtained. Blue STO 350, 400, and 450 show blue STO powders after reacting with NaBH_4_ at 350, 400, and 450 °C, respectively, and washing with water and ethanol. It is clear from Figure 1 that by tuning the temperature, the oxygen vacancy concentration can be tailored.

Consequently, the white color of pure STO can be turned to dark blue after heat treatment with NaBH_4_. The unreacted NaBH_4_ was washed easily with water and ethanol; the light blue color of STO was obtained after washing. It should be noted that increasing annealing temperature shifts the color of samples into darker regions as a result of the increase in the absorption band and oxygen vacancy concentration on the surface of STO.

**Figure 2:**
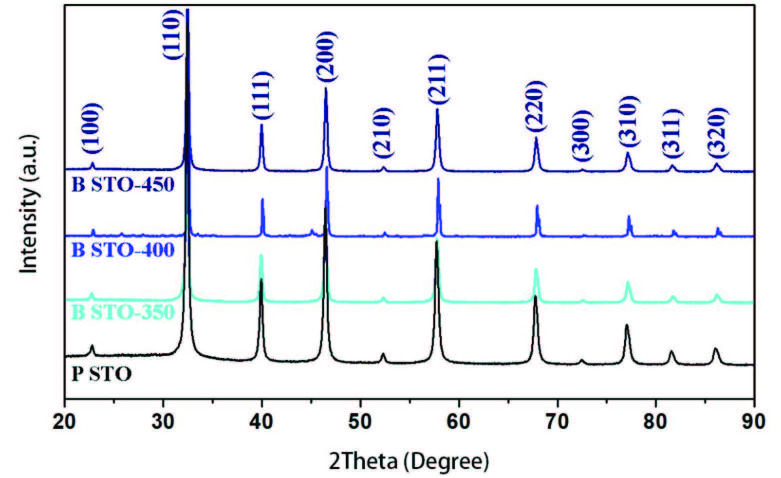
XRD patterns of pure STO and blue STO samples annealed at different temperatures.

Figure 2 shows the XRD patterns of pure and blue STO samples. From Figure 2, the reflected peaks at 2θ = 22.5, 32.6, 40.2, 46.5, 52.4, 57.9, 69.8, 77.5, 82.2, and 86.7 are ascribed to the cubic phase of pure STO perovskite (JCPDS 84–0443). Regarding the sharp peaks, it is clear that high crystallinity and pure STO was obtained without any SrCO_3_ impurities. Besides this, there are no apparent differences between the XRD patterns of the pure and blue STO. Although the phase structure of pure STO is not affected by treating with NaBH_4_, it seems that the peak positions, especially at higher 2θ are shifted to higher values because of increasing oxygen vacancy concentration.

**Figure 3:**
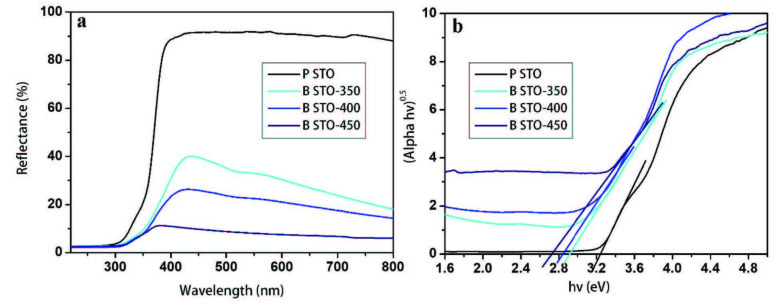
a) Diffuse reflectance spectra and b) calculated Tauc plots showing band gap of pure and blue STO annealed at different temperatures.

Figure 3 shows the UV-vis diffuse reflectance spectra of pure and blue STO samples and the related Tauc plots of their reflectance data. From Figure 3a, it can be seen that the diffuse reflectance of blue STO samples shows a broad absorption at wavelengths above 400 nm, which is probably caused by the trapped charge carriers or the absorption of incident light by oxygen vacancies of blue STO samples. Thus, it is not surprising that blue STO samples display more absorbance in the visible region than the pure one. Additionally, increasing the annealing temperature enhances absorption since the oxygen vacancy concentration increases accordingly. The bandgap calculation was performed through a procedure known as the Tauc model from diffuse reflectance data according to the following equation:

(hνα)^1/n^ = A(hν – E_g_)

where h, ν, α, A, and E_g_ are Plank’s constant, the frequency of vibration, absorption coefficient, the proportional constant, and bandgap, respectively. In this equation, n depends on the band structure of the sample; and therefore, it is 2 for an indirect allowed transition (n = 2). By using diffuse reflectance data and the Kubelka–Munk function (α = (1−R)^2^/2R, R is the reflectance), (hνα)^1/n^ can be plotted against hν (Figure 3b). Extrapolating the plot to the horizontal axis gives the bandgap (E_g_) value [33]. As seen from Figure 3b, it is interesting that the self-doping of STO leads to a drastic reduction in the energy bandgap. Based on the results, narrowing the bandgap with self-doping leads to diminishing the bandgap of pure STO from 3.2 eV to 2.9, 2.81, and 2.6 eV for blue STO annealed at 350, 400, and 450, respectively. This trend indicates that surface oxygen vacancies of blue STO were incorporated into the crystal lattice; consequently, they reduced the bandgap from 3.2 to 2.6 eV and extended the absorption to a longer wavelength range. Moreover, the red-shift can be apparently seen with increasing annealing temperature, and it would improve the UV-vis photocatalytic activity. 

**Figure 4:**
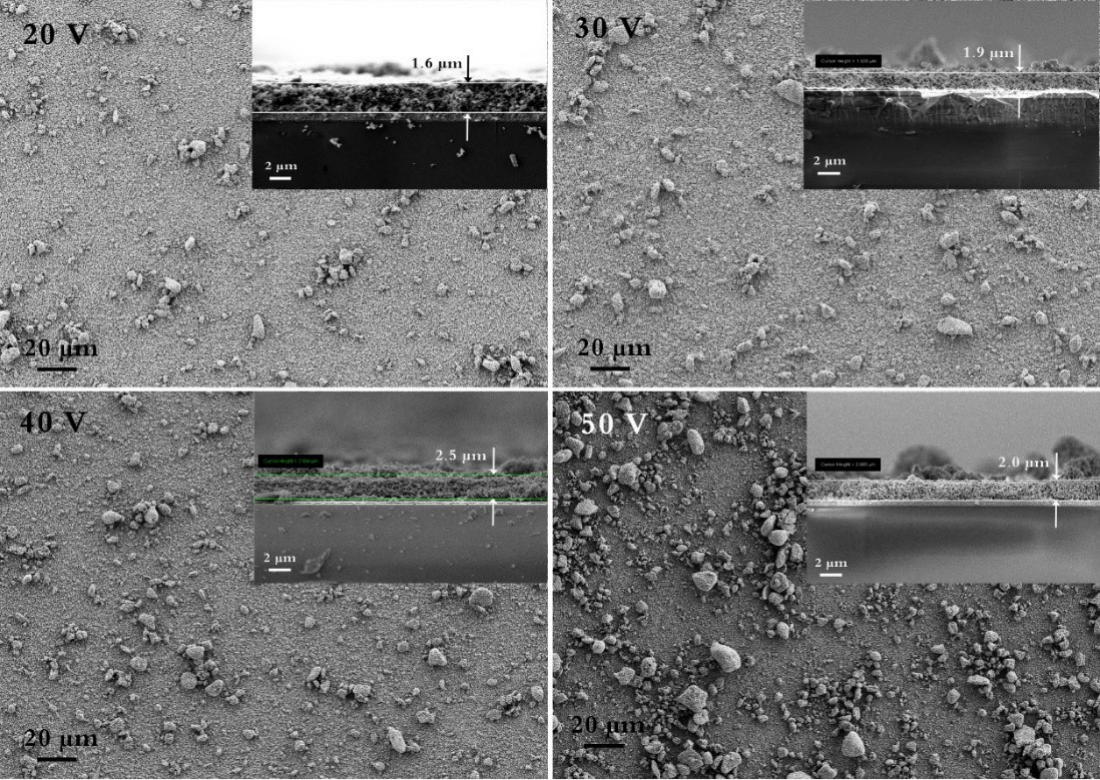
FESEM images of the top-view and cross-section morphology of STO powder deposited at different voltages of 20, 30, 40, and 50 V

The electrophoretic deposition was used to deposit STO powder on FTO conductive substrate. This deposition method produced transparent, pure, and blue thin film STO on FTO. Different voltages and times were applied to receive thin film with high uniformity. Figure 4 illustrates FESEM images of the top-view and cross-section morphology of STO powder deposited at different voltages, including 20, 30, 40, and 50 V. As seen from Figure 4, although raising the voltage results in layer thickness growth, it has an adverse effect on the uniformity of coating. The FESEM images reveal that agglomeration on top of the surface layer increases with increasing applied voltages. To this end, to get a smooth and uniform STO thin film by electrophoretic deposition, lower voltages are quite practical and can be chosen.

**Figure 5:**
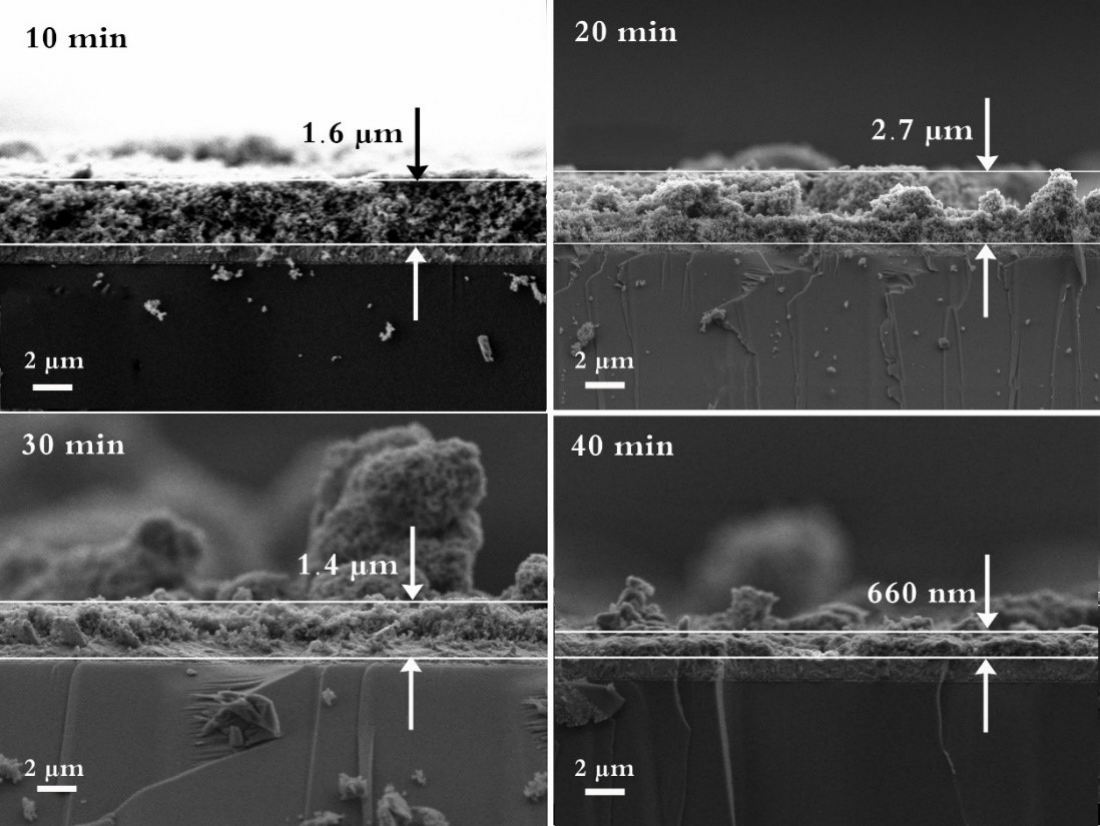
FESEM images of the cross-section morphology of STO powder deposited at different time limits of 10, 20, 30, and 40 min for a duration of 10 min.

Figure 5 depicts FESEM images of the cross-section morphology of STO powder deposited at different time limits of 10, 20, 30, and 40 min. For these samples, the electrophoretic deposition was performed at the voltage of 20 V. It is evident from Figure 5 that the uniformity of the STO layer was lost with increasing the electrophoretic deposition time, and the thickness of the layer was reduced. In fact, increasing the deposition time would not help to improve the uniformity of the top layer. As a result, based on the electrophoretic optimization results, the voltage of 20 V and time of 10 min was selected for obtaining uniform transparent coating of STO on the FTO substrate.

**Figure 6:**
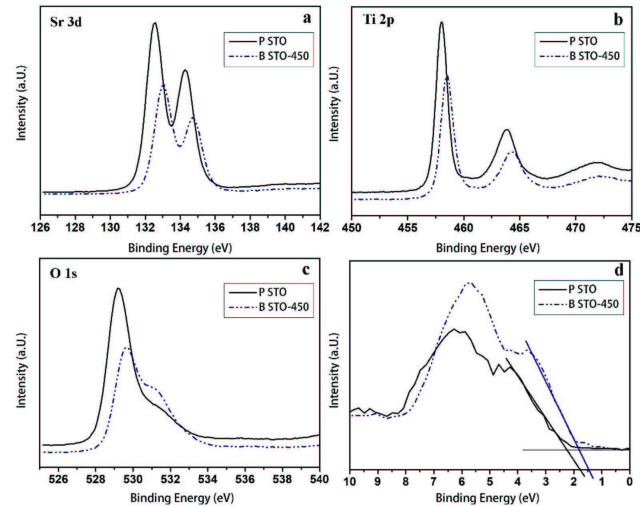
a) Sr 3d XPS spectra, b) Ti 2p XPS spectra, c) O 1s XPS spectra, and d) XPS valence band spectra of pure STO and blue STO-450 samples.

Figure 6 shows the high-resolution XPS spectrum of Sr 3d (Figure 6a), Ti 2p (Figure 6b), O 1s (Figure 6c), and the valence band position of pure STO and blue STO-450 coatings on FTO substrates (Figure 6d). Concerning the XPS results, it can be concluded that in blue STO-450, the binding energies of Sr 3d, Ti 2p, and O 1s are shifted to higher binding energies. These shifts are as a result of the presence of Ti^3+^ and oxygen vacancies in blue STO in comparison to the pure one. The oxygen vacancy increases the strength of the equilibrium electron density and consequently pushes the Fermi level upward. The valence band position of pure STO and blue STO-450 samples are shown in Figure 6d. The pure STO manifested the valence band edge at around 1.8 eV below the Fermi level. As the bandgap of pure STO is about 3.2 eV (Figure 3b), the conduction band minimum is most likely located at about –1.4 eV. On the other hand, for the blue STO-450 sample, the valence band position displayed a blue-shift toward 1.4 eV, lower binding energy, which may be attributed to the surface oxygen vacancies disorderliness of blue sample. The uplift of the valence band is due to disorder shell in blue STO structure. The reduction of the bandgap of blue STO-450 to 2.6 eV (Figure 3b) is related to both the uplift of the valence band (due to surface disorderliness), and the lowering of the conduction band of blue STO, in consequence of the presence of oxygen vacancy defects and Ti^3+^ energy level formation below the conduction band. 

**Figure 7:**
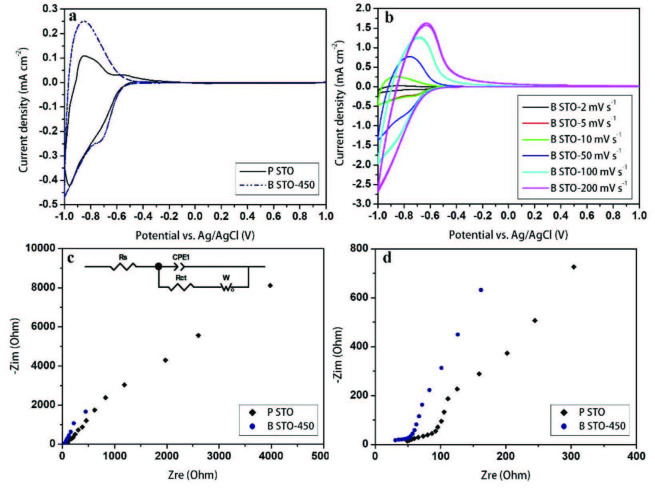
a) CV curves of pure STO and blue STO-450 samples, b) CV curves of blue STO-450 sample measured at different scan rates, c) and d) Nyquist plots measured at OCP in the supporting electrolyte of 0.1 M KH_2_PO_4_.

To determine the electrochemical properties of pure STO and blue STO-450 samples, and to evaluate the effect of NaBH_4_ on the electrochemical properties of blue STO, cyclic voltammetry (CV) measurements were conducted on the pure and blue STO in 0.1 M KH_2_PO_4_ solution. Figure 7a shows the CV curves of pure and blue STO samples at a scan rate of 10 mV s^-1^. The triangle shape of the CV curves displays an n-type semiconducting behavior of the samples. From Figure 7a, the current density of the blue STO-450 sample reveals higher values than the pure STO indicating low resistance and high electron mobility of blue STO. The presence of oxygen vacancy and high level of Ti^3+^ states reduce electron mobility resistance of the sample, and thereby low resistances can enhance the current density. In addition, from Figure 7a, the H_2_ evolution peak can be seen in the blue STO sample around negative voltage of –0.7 V. Figure 7b shows CV curves of blue STO-450 recorded at different scan rates of 2–200 mV s^-1^ indicating the capacitive behavior of blue STO. As depicted in Figure 7b, the triangle shape of the curves remains intact even at a high scan rate of 200 mV s^-1^. 

To precisely explore the self-doping effect of blue STO on electrochemical behavior, EIS measurements were performed at an open-circuit voltage to predict charge transfer resistance through the electrodes. Figures 7c and 7d present the Nyquist plots and the equivalent circuit of the pure STO and blue STO-450 electrodes in full and zoom screen, respectively. The Rs is the solution resistance between the working and reference electrodes. Regarding the fact that diffusion is the controlling step at the interface, a series combination of Faraday resistance and Warburg impedance is considered. Thus, the constant-phase element (CPE) is replaced by the capacitive element due to the complex nature of the interfacial reaction.

It can be seen from Figures 7c and 7d that the Nyquist plots consist of a line at 45° at high frequencies followed by a vertical line at low frequencies. A line at 45° illustrates the identical behavior of the real and imaginary parts. Moreover, according to the shape of the Nyquist plot, diffusion is considered a reflective boundary finite-length linear (the diffusion layer corresponds to the layer thickness), because the plot does not include the entire arc region and the sample is not strictly capacitive [33]. A straight line at 45° can be observed in the mass transfer impedance at high frequencies because the penetration length of the AC signal is smaller than the layer thickness. On the other hand, at lower frequencies, the constant current cannot flow in the electrode, the imaginary part goes to infinity and the electrode displays capacitive behavior. Therefore, the finite-length reflective impedance is considered a Warburg element (open) (Wo) in ZView interpreting software. The Warburg impedance can be detected as a linear Nyquist plot at the mid- and low-frequency regions.

The effect of the oxygen vacancies can be interpreted from the data shown in Figures 7c and 7d. Table shows the calculated values of the equivalent circuit elements for the electrodes. From Table, the interface charge transfer resistance (Rct) of blue STO was reduced to 6.38 Ω cm^-2^. It can be concluded that in the blue STO electrode, the finite-length diffusion becomes completely capacitive due to the reduction of the bandgap as a result of the oxygen vacancy levels. Therefore, the reducing process significantly improves the electron transport conditions of the blue STO sample compared to the pure one. Besides, the Warburg resistance of blue STO reveals a very astonishing diminution down to 1 Ω cm^-2^.

**Table:** The calculated values of the equivalent circuit elements for the electrodes.

Sample	Pure STO	Blue STO-450
Rs (Ω cm–2)	52.3	11.4
CPE1-T (mF Sp–1 cm–2)	6.67 × 10–6	1.25 × 10–5
CPE1-P	0.45	0.72
Rct (Ω cm–2)	84.2	6.38
W-R (Ω cm–2)	893.6	1.154
W-T	2.21 × 10–6	5.58 × 10–5
W-P	0.39	0.46

## 4. Conclusion

In summary, the Ti^3+^ self-doped SrTiO_3_ with a reduced bandgap was prepared via reaction with NaBH_4_. The resulting blue SrTiO_3_ shows high crystallinity without any impurity and oxygen vacancy concentration in the structure. In this work, the bandgap was reduced from 3.2 eV for pure SrTiO_3_ to 2.6 eV for blue SrTiO_3_; therefore, absorption was extended to a long wavelength range. The electrophoretic deposition was successfully applied to deposit SrTiO_3_ powder on the FTO conductive substrate. The electrophoretic voltage of 20 V and a time limit of 10 min were optimized to reach evenly uniform coatings on FTO. Electrochemical studies showed that the presence of oxygen vacancy and high level of Ti^3+^ states reduce electron mobility resistance and therefore lead to a higher current density of 0.25 mA cm^-2^ compared to pure SrTiO_3_. Based on electrochemical results, it can be concluded that in the blue STO electrode, the finite-length diffusion becomes completely capacitive due to the reduction of the bandgap, stemming from the oxygen vacancy levels. Therefore, the reducing process significantly improves the electron transport conditions of the blue STO sample compared to the pure sample, and blue SrTiO_3_ exhibits lower resistance (charge transfer resistance of 6.38 Ω cm^-2^). Furthermore, the Warburg resistance of blue STO reveals a dramatic diminution down to 1 Ω cm^-2^.
